# Psychometric properties of the Greek translation of the Greek Short Dark Triad questionnaire (GSD3Q) in a subclinical sample

**DOI:** 10.1192/j.eurpsy.2023.424

**Published:** 2023-07-19

**Authors:** G. N. Lyrakos, E. Aslani, V. Spinaris

**Affiliations:** ^1^Psychiatric, General Hospital Nikaia “Agios Panteleimon”, Nikaia; ^2^Psychology, City Unity College, Athens; ^3^2nd Department of Pathology, General Hospital Nikaia “Agios Panteleimon”, Nikaia, Greece

## Abstract

**Introduction:**

‘Dark Triad’ is a constellation of three conceptually distinct but empirically overlapping personality variables. The three members- Machiavellianism, narcissism and subclinical psychopathy – often show differential correlates but share a common core of callous-manipulation.

**Objectives:**

To validate an easy to use and valid measurement for subclinical populations examining the 3 factors of the dark triad of personality.

**Methods:**

A pool of items designed to circumscribe the classic conceptions of the Dark Triad constructs used for the Short Dark Triad were translated using the multiple forward and backward translation protocol. Participants consisted of 391 adults recruited from social media. Participants were then asked to rate their agreement with each of the 44 items generated for the GSD3Q).

**Results:**

391 adults participated, 51 (13%) male, 340 (87%) female mean age 41.4(SD=10.8). Corrected Item-Total Correlation was used for the first reduction of items and then PCA with eigenvalues >1.5 was used for the reduction of the items loading lower than 0. 400 for each factor independently. Finally, Principal Axis Factoring with Varimax rotation led to 9 items for Machiavellianism (alpha coefficient=0.659), 8 items for Narcissism (alpha coefficient=0.659) and 10 items for Psychopathy (alpha coefficient=0.742). Strong correlations were found as expected between the 3 factors with lower between Machiavellianism and Narcissism r=.487 p=0.001 and higher between Psychopath and Narcissism r=.617 p=0.001.

**Conclusions:**

The results of the current validation led to a 27 item, with 3 factors questionnaire with similar psychometric properties for non-clinical populations. Further validation is needed for clinical samples.

**Disclosure of Interest:**

None Declared

